# A novel fasting regimen revealed protein reservation and complement C3 down-regulation after 14-day’s continual dietary deprivation

**DOI:** 10.3389/fendo.2023.1150547

**Published:** 2023-07-06

**Authors:** Yaqian Hao, Yu Fu, Liangliang Sun, Yaying Yu, Xia Min, Qiannan Wei, Shuangjian Huang, Sen Zhao, Li Wang, YuanYuan Wang, Yangyang Li, Xia Zheng, Chenlu Zhang, Hongxia Xu, Xiaoxue Wang, Garrick D. Lee

**Affiliations:** ^1^ The First Affiliated Hospital of Henan University, Kaifeng, Henan, China; ^2^ College of National Security, University of National Defense, Beijing, China; ^3^ Institute on Aging and Disease of Henan University, Kaifeng, Henan, China

**Keywords:** Flexible Abrosia, continual dietary deprivation, metabolic inflammation, myostatin, complement C3

## Abstract

**Objectives:**

The aim is to evaluate the effect of a novel 14-day fasting regimen on the balance between skeletal muscle and adipose tissue composition which might associate with inflammatory factors. Our analysis includes basic physical examinations, clinical laboratory analysis, bioelectrical impedance and biochemical analytic assessments of healthy volunteers.

**Methods:**

Eight healthy subjects were randomly selected from a pool of volunteers to undergo a continual dietary deprivation (CDD) regimen. Individuals were assigned to take Flexible Abrosia (FA, prebiotic combination) plus appropriate mineral supplement of potassium and magnesium at 3 mealtime every day to prevent potential injury from starved intestinal flora and avoid spasms of smooth muscle due to hunger. Physical and medical examinations were conducted and blood samples were collected at following timepoints: before CDD as self-control (0D), day 7 and day 14 during fasting, and 7-21days and/or 2~3mo after refeeding.

**Results:**

The combination of FA and mineral supplements significantly decreased self-reported physical response of starvation, with tolerable hunger-mediated sensations experienced during CDD. Bioelectrical and biochemical results indicated significant reduction in both muscle lean and fat mass on day 7. Meanwhile, markers related to fat composition consistently decreased during and after CDD. In addition, most biochemical marker levels, including serum proteins, reached their inflection points at the 7th day of CDD as compared to the control measurements. Levels of these factors started to show a relative plateau, or reversed direction upon the 14th day of CDD. The exceptions of above factors were myostatin and complement protein C3, which remained at lower concentrations in the blood throughout CDD, and were unable to fully recover toward baseline levels even after 3 months’ refeeding.

**Conclusion:**

Our results indicated that human subjects undergoing prolonged dietary restriction were well protected by FA and mineral ions from gut injury or physical discomfort of starvation. Most factors showed a relative plateau response at the end of 14D-CDD. The muscle tissues were well preserved during prolonged fasting, and an improved protein/lipid ratio was observed. Upon refeeding, constant lower levels of myostatin and complement C3 were maintained after CDD implies a long-term beneficial effect in dealing with anti-aging and inflammation.

## Background, motivation, and objectives

Under certain conditions, fasting regimens were shown to be more effective and efficient in achieving health benefits than other types of dietary interventions including dietary restriction (1). Evidence from modern studies of fasting demonstrated potent lifespan extension in lower species, such as *E. coli*, yeast, and *C. elegans (* 2–5). Up to date, fasting in humans has mostly been studied in the context of short-term or intermittent schedules, as they are safer and more practical applications which were initially published by BUCHINGER (6). Another fasting regimen mentioned in the literature include very low calorie (200~500 kcal/day) fasting up to a year in duration for weight management, disease prevention and chemotherapy facilitation (7–9). Under the current schedules of both alternative fasting or longer-term CR, individuals must undergo months or even years of low-calorie dieting in order to obtain a significant anti-aging benefit, and sometimes negative consequences from this long-term deprivation of energy supply can impact quality of life.

Prolonged fasting has been extensively studied last century, which have carefully approached physiological responses and metabolic consequence in human under extreme status of starvation (9, 10). It was reported that the concentration of valine doubles in the plasma during the first 7–10 days of starvation, and then slowly and progressively falls to a value below its overnight fasting value. Venous glutamine, the predominant amino acid in plasma, remains relatively stable during 5–6 weeks of fasting in human (9). Although varied duration and types of starvation have been approached, seldom of the regimen has successfully applied as practical protocol in society. Based on our previous work involving prolonged fasting, we have confirmed that our reported regimen, Flexible Abrosia (FA, with 113.4 KJ in each 10-g pack) which has been reported as FA-CDD administered for 7 days (11), has been shown to serve as an alternative nutrient supplement for gut microbiota and prevent them from damaging the colonic mucus barrier due to consuming glycoproteins during host fasting (12).

Considering the life-threatening complications during fasting for more than 40 days (13–15), we chose to conduct our study for a more practical fasting period of 2 weeks as maximum for the entire CDD treatment. Our previous work also demonstrated the changing patterns of protein and lipid concentrations in plasma, and found that concentrations of protein were favored after fasting for 7days (11). Therefore, in the current study, we limited the maximum fasting time to no more than 14 days, before the slope of body weight loss became varied (9, 16). We designed the experiment to reduce the starvation experience and further explore the relationship between muscle and fat under a specially designed prolonged fasting paradigm. Based on the promising findings of our first study of 7D-CDD paradigm (11, 17), we have enrolled eight subjects to focus on observing the effects of FA-facilitated prolonged fasting of 14D-CDD. Our report supplies evidence in evaluating the physiological and biochemical effects of this fasting regimen, and will discuss the different pattern of changes observed in both muscle (protein) and fat (lipid) under the impact of oxidative stress and inflammatory factors observed in our novel prolonged fasting regimen.

## Materials and methods

### Study design and participants

A complete clinical trial registration has been deposited with the Chinese clinical trial registration organization (http://www.chictr.org.cn with registration # ChiCTR-OOC-17010377). Approval of the study protocol was given by the University of Henan Human Research Protection Program under the guidance of the China Association for Ethical Studies. The protocol was also documented with the Medical Ethics Committee of Henan Medical Association of Henan Province, and the entire clinical study was conducted under the supervision of the ethics board of the hospital as described previously (11). Before initiating the program, signed informed consent was obtained before participation, and history, physical, electrocardiogram, laboratory, physical, and ultrasound exams were also performed as pre-med checks. Inclusion criteria were as follows: (i) volunteers from the hospital staff, including doctors, nurses, lab and medical technicians, etc., and their relatives, (ii) age 21–65 years, (iii) absence of any exclusionary factors among the individuals participating, such as active medical or psychiatric problems, history of heart disease and potential heart problems such as heart failure, myocardial infarction, and cardiac arrhythmia, renal dysfunction, serious blood clots, intestinal obstruction or ulcer, or type-1 diabetes patients with islet dysfunction.

### Procedures

Volunteers were recruited and introduced to the FA-CDD program. We have initiated a new protocol with application of prebiotics in dealing with prebiotics based on previous human starvation studies of the past century (11). We have later targeted to further relieve the suffering of long-term starvation by application of herb subtracts for release smooth muscle spasm and mineral supplies in order to supply more practical paradigm for the society. The current set of the results were obtained through the initial attempt of the protocol which have been demonstrated applicable, and the subsequent results from improved protocol would be further conducted and discussed in subsequent reports in the near future. In order to simplify the sophistication of the influence by geographical distribution, ethnicity and gender, we focused our study only on the central plain regions of China with 5 female and 3 male subjects. Before the initiation of the trial, the individuals received medical and laboratory examinations, including collections of serum, plasma, urine, and feces samples as described previously (11). FA-CDD related with daily oral consumption of solid beverage for Flexible Abrosia (FA) at 10g/day/person at three meal times during fasting. The ingredients of FA were designed to include dietary fiber and cordyceps polysaccharide, ganoderma lucidum polysaccharide, and hericium erinaceus polysaccharide, which were regarded as bacteria but no human-consumable saccharides, which reported to contain energy of 113.4 KJ (27 kcal) per 10g FA, which indicated that even if the calories from each treatment were completely absorbed by the human being as previously reported (11), the calorie intake would be still lower than previously reported alternative dietary supply (18, 19). During CDD, subjects were under the outpatient observation through group online guidance and supervision, which were fully supported by experienced hospital staffs 24hours per day. The attendance of the hospital staffs would be available 24hours/day during the observation. Individuals were advised to avoid any food intake, especially carbohydrates, except to drink plenty of water and take FA, mineral electrolyte and vitamin.

During the first 3~5 days, to overcome symptoms such as stomach discomfort and cravings for food, individuals were allowed to consume a few pieces of low-sugar fruit or vegetables such as cucumber or tomato, or ingest a minimal amount of lipids with a few peanuts or dried beef. Further, during periods of extreme hunger in the day time, participants could also consume either 1 stick of 375 mg potassium/400 mg magnesium (DAS Gesunde Plus, Deutschland) or 1 pellet of 1.08 g (K=10 mEq) potassium citrate extended-release tablets (Dawnrays China), which further ameliorated fasting-induced mineral loss and reduced peristaltic pangs of the smooth muscles of the gut. During the later period of CDD, individuals usually adapted to the physical experience of starvation and would be able to handle the physiological 170 symptoms of starvation easier. According to the written informed consent forms, the individuals could withdraw from the ongoing program at any time and at any step of the experiment without giving an explanation.

During the entire experimental fasting period, participants were asked to record any observed symptoms of starvation and overall fasting in a questionnaire supplied by the project administrators. Hunger sensation was reported among 4 of participants in numeric ranking from 1-10 as following:

1) Normal daily life in *ad libitum*;
2) Digestive tract is comfortable and the body is relaxed;
3) Slight hunger feeling, wish to eat something;
4) Obvious feeling of hunger and fatigue. The subject starts to feel certain intense pressure in stomach, but can choose not to eat;
5) Tolerable hunger and fatigue, obvious tension in stomach, but can resist eating;
6) Hunger and fatigue getting stronger and stronger. It takes a little perseverance to continue, but one can still resist eating;
7) Hunger and fatigue becoming more and more intolerable, and the desire to eat more prominent;
8) Hunger and fatigue becoming very strong, and showing irritable temperament and strong desire to eat;
9) Hunger and fatigue more and more intense and intolerable, feeling the pressure of twitching and contracting in gut. Intense desire to start to eat;
10) Hunger and the willingness to search for food extremely strong, with severe contraction of gastrointestinal tract. Feel unable to adhere to the protocol and decide to quit.

Physical and neurological examination, weight, blood and urine chemistries, electrocardiogram, bioelectrical impedance directed body composition analysis were performed at following five time-points by certified medical authorities of the hospital: 1) before the regimen initiated (control baseline- *ad libitum*); 2) 7^th^ day of fasting; 3) 14^th^ day of fasting; 4) after recovery with food intake for 7-21 days, 5) after recovery with food intake for 1-3 months. Due to the difficulty in accurately performing samples collection and physical exam, we grouped refeeding 7-21D’s samples as Refeed 7-21D, and 21-90D’s refeeding as Refeed 1-3M.

Bioelectrical impedance analysis (BEIA) was performed using the InBody 720 Body Composition Analyzer. The InBody 720 has received FDA approval for analysis of impedance, reactance, and resistance in measuring body composition. The instrument is able to assess body water, protein, muscle, mineral, and fat content, and more, rather than just measuring Body Mass Index (BMI). The device can determine the weight of lean muscle tissue in each limb, water content, percentage body fat, mineral content, protein content, and visceral fat levels. Measurements at five timepoints during the CDD experiment data were supplied via a full-color print-out and were examined by a professional analyst at the hospital as described previously (11).

### Biological sample analysis

The medical and basic laboratory exams were performed at The First Affiliated Hospital of Henan University. We also performed the following molecular and biochemical tests on biological samples collected during the CDD experiment:

1) Plasma or serum preparations. Plasma or serum was centrifuged at 3000 rpm and analyzed according to hospital protocol.
2) Serum factor measurements. TNF-α(E-EL-H1205c), human glucagon (E-EL-H2237c), myostatin (E-EL-H1437c), and Insulin-like growth factor 1 (E-EL-H0086c) levels in serum were detected through ELISA kits (Elabscience Biotechnology Co., Ltd., Wuhan, China). The ELISAs test were performed at Beijing Institute of Radiation Medicine.

### Statistical analysis

Statistical analyses were performed using JMP, version 10.0 (SAS Institute), and GraphPad Prism 8.0.1 (1992-2016 GraphPad Software, Inc). One-Way ANOVA with repeated measures (with Tukey’s multiple comparisons test) was applied to data from 8 individuals and plotted in GraphPad Prism. We also reported multiple t-test comparisons between fasting and after refeeding procedures. For some individual, the refeeding procedure could not be strictly followed on exact time interval. We then rounded the specific refeeding time points such as refeeding 7-21D and 1-3M as average. For some special factors, such as CK and myostatin, we compared the value of refeed 7-21D or 1-3M with each subject’s own control and tested by Student’s paired t-test (continuous variables).

## Results

### Physiological and bioelectrical impedance analysis results

**Figure 1:**
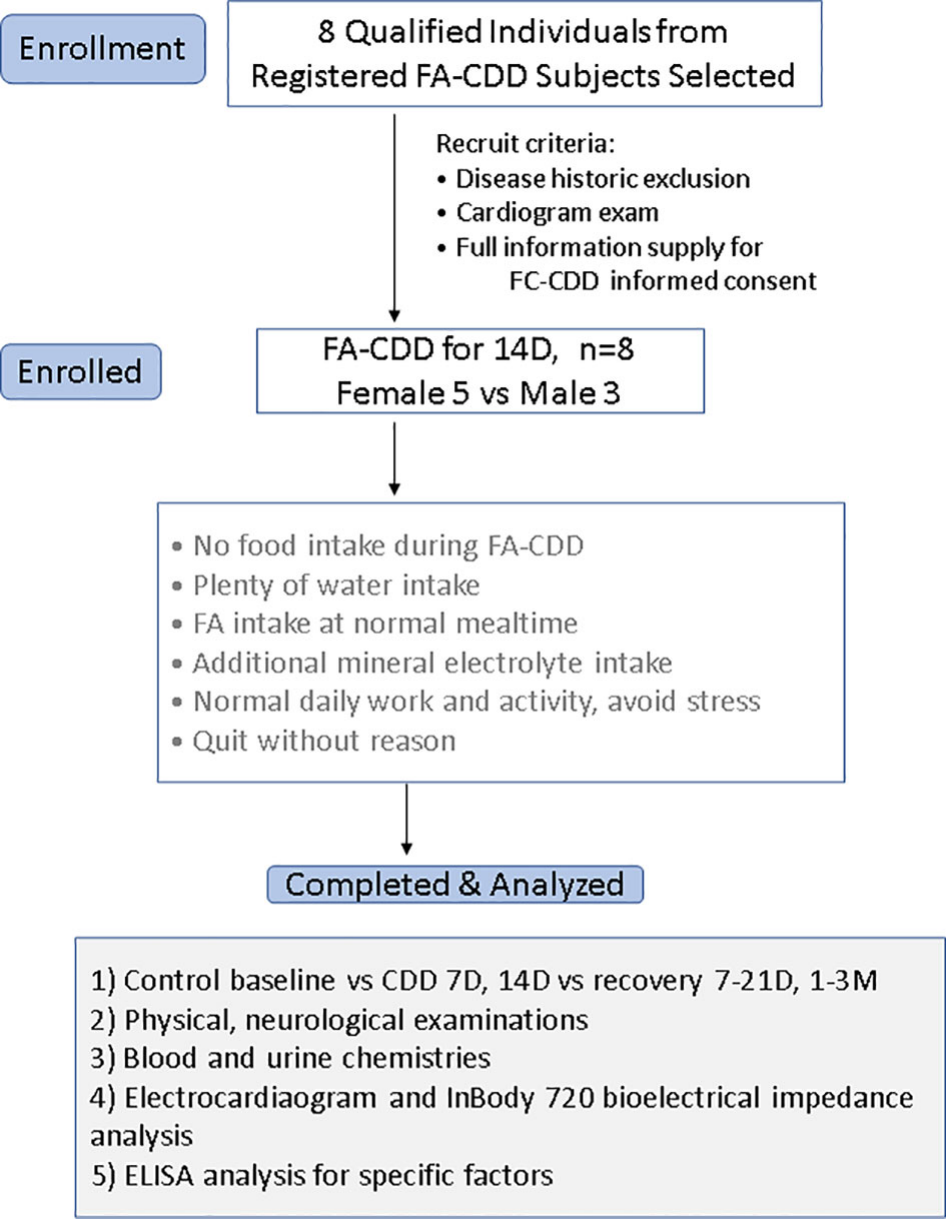
Flow chart of the clinical observation procedure. Analyses of 14D-CDD were basing on 8 subjects (3 men and 5 women) and were using their own before CDD baseline as ad libtum control.

**Figure 2:**
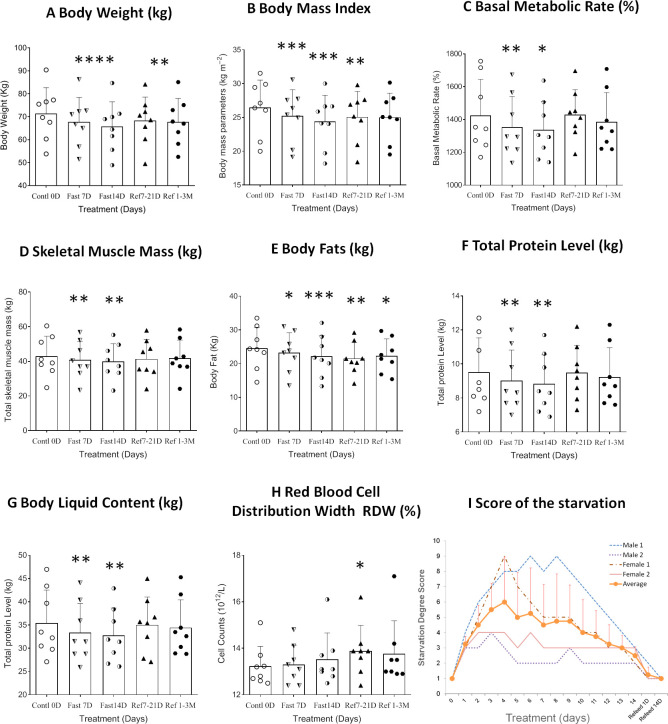
Differential analysis among 0D, 7D, 14D fasting, and refeed recovery 7-21D, 1-3M results from clinical laboratory tests. Data were represented as MEAN ± SD in physical examination, physical bioelectrical impedance analysis, and self-estimation results with same range of treatment days in all experiment. The entire 14D’s fasting followed by 7D to 3mo’s refeeding were analyzed using repeated measure of one-way ANOVA on each column of variable. X-axles refer to control 0D, fasting 7D &14D groups, refeeding 7D &50D treatment groups (refeeding groups were assorted with recovery within 14D as refeed7D, and more than 21 days’ group as refeed50D due to the uneven data collections after CDD treatment), respectively. Graph were created with GraphPad Prism 8.0.1 software using one-way ANOVA with repeated measures, and the dots around each column represent the actual values of group subjects on the specific treatments. Each treatment was analyzed with Tukey’s multiple comparisons test. Significance was assigned according to the description of GraphPad Prism: * p<0.05, ** p<0.01, *** p<0.001, **** p<0.0001; the actual values are represented in the correspondent tables. **(A)**. Weight change records during 14D-CDD fasting. Since standard deviation was close to 10kg for each date point, and the body weight ranges shown in the Y-axis is less than 10kg, we excluded error bars to keep the graph clean. **(B–H)**. Data were represented as results in MEAN ± SD of Bioelectrical impedance analysis (BEIA). **(I)**. Summary of score of the starvation was based on individual’s own evaluations of every day’s experience (n=4, Male 2 and Female 2). The average evaluation was represented as MEAN ± SD but only recorded during the fasting period (1~14D).

Eight healthy subjects who had successfully completed a 14D-CDD trial with the concomitant administration of FA were observed throughout the duration of their fasting and their medical conditions were strictly monitored by hospital medical experts (**Figure 1**). Our results indicated that the body weight (BW) and body mass index (BMI) were reduced slightly faster in 7D-CDD than 14D-CDD and that the refeeding after CDD failed to return these parameters to the baseline levels completely (**Table 1** and **Figures 2A, B**). Meanwhile, the basal metabolic rate (BMR) was also reduced at 7D-CDD, and the level remained similar at 14D-CDD; interestingly refeeding resulted in a rebound of BMR but not of body weight (**Table 1** and **Figure 2C**).

**Table 1:** Bioelectrical impedance analysis (BEIA) results under Flexible Abrosia facilitated Continual Dietary Deprivation for 14 days.

	Control 0D	Fasting 7D	Fasting 14D	Refeed 7-21D	Refeed 1-3M	One-way ANOVA F, *p* value
**Weight (kg)**	71.39±11.27	67.79±10.62 ****	65.69±10.85 ****	68.26±10.29 *	67.75±10.18 *	**14.52, p=0.0003**
**BMI (kg m^–2^)**	26.48±4.05	25.23±3.86 ***	24.40±3.87 ***	25.08±3.76 **	25.03±3.57	**12.89, p=0.0013**
**Basal metabolic rate (%)**	1424.17±220.00	1353.97±186.13 **	1336.64±174.15 *	1429.56±151.82	1385.45±178.32	**5.78, p=0.0198**
** **						
**Total skeletal muscle mass (kg)**	42.98±11.57	40.78±10.74 **	39.99±10.22 **	41.43±11.10	41.71±10.56	3.26, p=0.0589
**Trunk muscle mass (kg)**	21.68±4.33	20.56±3.96 **	20.07±3.80 **	21.51±3.62	20.84±3.84	**5.69,p=0.0177**
** **						** **
**Body fats (kg)**	24.59±6.21	23.28±5.87 *	22.18±6.17 ***	21.53±4.73 **	22.33±5.00 *	**9.19, p=0.0015**
**Body fat percentage (%)**	33.63±5.69	33.49±5.58	32.87±5.91	30.69±3.59	32.07±4.94	3.66,p=0.0561
**Degree of whole body obesity (%)**	123.26±17.19	117.36±17.01 ***	113.94±16.26 ***	117.44±14.79 *	117.65±14.578	**12.82, p=0.0016**
**Visceral fat area (m^–2^)**	114.10±39.51	107.95±38.60 *	102.98±39.67 **	95.18±30.63 *	100.47±35.71 **	**7.03, p=0.0052**
						** **
**Total protein level (kg)**	9.51±2.02	9.01±1.80 **	8.83±1.71 **	9.48±1.60	9.23±1.73	**4.33, p=00332**
**Body liquid content (kg)**	35.41±7.12	33.35±6.28 **	32.79±5.87 **	35.05±5.99	34.43±6.01	**3.98, p=0.0465**

Comparing different stage of CDD treatments with Control 0D under Dunnett's multiple comparisons test.

Significance * p<0.05, ** p<0.01, *** p<0.001, **** p<0.0001, Sample size=8. Bold number indicated the detail significance of the value.

Bioelectrical impedance analysis (BEIA) results indicated that muscle-related parameters (skeletal muscle mass and trunk muscle mass) decreased at slower speed at the 14^th^ day of CDD than the 7^th^ day, and it recovered quickly after the CDD was completed (**Table 1** and **Figure 2D**). This is in contrast to fat-related parameters (ratio of overall body fat to visceral fat area), which maintained a pattern of constant decrease during both fasting and refeeding periods. These results might imply that human metabolism under fasting conditions may utilize lipid metabolism more efficiently than muscle catabolism once the metabolic equilibrium is established after longer-term fasting (**Table 1** and **Figures 2D, E**). Upon assessing protein levels through BEIA, it seems that the whole-body total protein level changes in a similar pattern to that of muscle mass, along with the parallel reduction of body liquid levels (**Table 1** and **Figures 2E–G**). These observations might explain the slower rate of reduction in BW during later stage of CDD (7D~14D) since the proportion of the body mass (relative stable at 14D in Total skeletal muscle mass and Total protein level; vs constant reduction in Body fats and Body fat percentage) might be different (**Table 1** and **Figures 2A–E**).

### Different metabolic levels of lipid and protein during and after fasting

**Figure 3:**
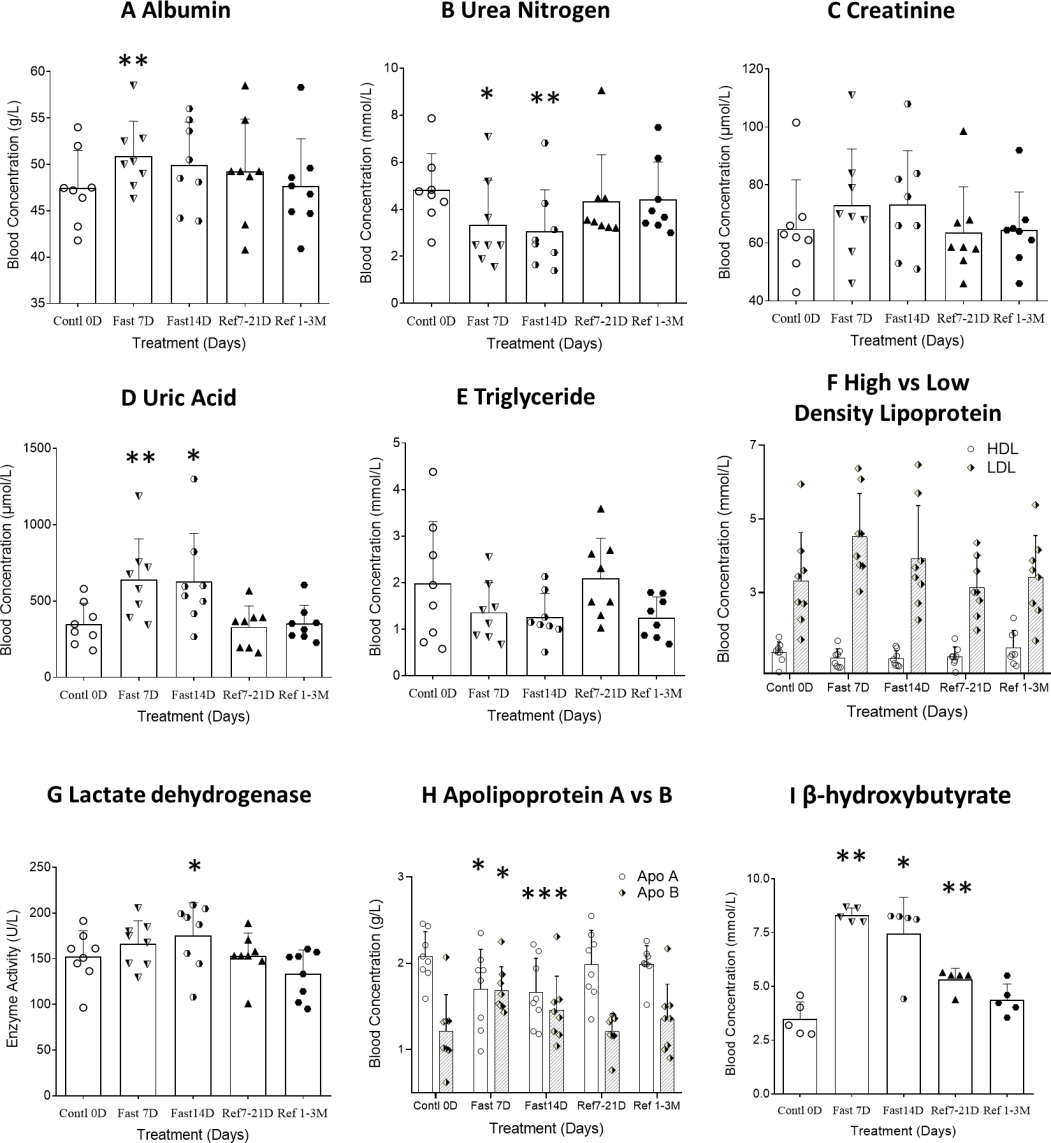
Differential analysis among 0D, 7D, 14D fasting, and refeed recovery 7-21D, 1-3M results from either clinical biochemistry tests or ELISA detection. Data were represented as MEAN ± SD in biochemistry and ELISA analysis with same range of treatment days in all experiment. The entire 14D’s fasting followed by 7D to 3mo’s refeeding were analyzed using repeated measure of one-way ANOVA on each column of variable. The data assignment is in the same was as described in **Figure 2**. Each treatment was analyzed with Tukey’s multiple comparisons test. Significance was assigned according to the description of GraphPad Prism: * p<0.05, ** p<0.01, *** p<0.001; the actual values are represented in the correspondent tables. **(A–H)**. Data were represented as results in MEAN ± SD of biochemistry analysis from hospital laboratory. **(I)**. Data were represented as results in MEAN ± SD of immunological analysis from ELISA analysis.

Based on the changing patterns of fat and protein composition observed by BEIA at different stages of the fasting regimen, we further analyzed basic routine clinical laboratory results to gain additional insight into metabolic perturbations. However, except for the volumes of both red blood cell (distribution width) and platelet count, no significant difference was observed in different blood cell types, hemoglobin or platelets (**Table 2** and **Figure 2H**). The behavioral analysis in reporting Score of the starvation indicated that different individual has different degree of hunger pang, which might be related with different physical status. In regarding with the biochemical results, although the blood concentrations of total protein, albumin and globulin increased at day 7 of CDD, all of them failed to further increase by day 14 of CDD. Albumin is associated with free fatty acid binding and transport throughout the body as an alternative source of energy supply. The significant increase of albumin at 7D-CDD might indicate an activation of alternative energy supply from hepatic glycogen breakdown. However, although this observation lacked statistical significance due to limited sample size, blood protein levels of volunteers also showed no further decrease at 14^th^D-CDD compared with 7^th^D-CDD, and the levels were stabilized during the refeeding recovery stage (**Table 3** and **Figure 3A**). Other protein related nitrogen metabolites, such as urea nitrogen, creatinine, and uric acid, showed some expected variation, but all showed nearly steady state metabolite levels between 7D and 14D-CDD (**Table 3** and **Figures 3B–D**).

**Table 2:** Laboratory hematology results under Flexible Abrosia facilitated Continual Dietary Deprivation for 14 days.

	Control 0D	Fasting 7D	Fasting 14D	Refeed 7-21D	Refeed 1-3M	One-way ANOVA F, *p* value
**Leukocyte count (×10^9^/L)**	6.87±2.06	6.28±1.25	7.25±2.60	5.43±1.30	6.81±2.31	1.92, p>0.1
**Neutrophil granulocyte count(×10^9^/L)**	5.20±3.10	3.78±0.99	4.62±2.59	3.34±0.80	4.189±1.80	1.32, p>0.3
**Lymphocytes count(×10^9^/L)**	2.24±0.70	1.94±0.40	2.07±0.54	2.01±0.43	2.04±0.64	0.75, p>0.5
**Monocytes count(×10^9^/L)**	0.43±0.16	0.43±0.16	0.45±0.11	0.36±0.11	0.45±0.13	1.70, p>0.2
**Eosinophilic granulocyte count(×10^9^/L)**	0.13±0.083	0.075±0.10	0.079±0.067	0.08±0.034	0.1±0.053	1.91, p>0.1
**Red blood cell count (×10^12^/L)**	4.78±0.60	5.17±0.66	5.02±0.79	4.84±0.49	5.11±0.84	1.21, p>0.3
**Hematocrit(L/L)**	1.06±1.70	0.45±0.061	0.86±1.15	0.42±0.042	0.45±0.078	0.79, p>0.4
**MCV (fL)**	87.04±4.03	87.11±3.61	88.33±4.18	87.43±3.25	87.94±3.95	1.65, p>0.2
**Red blood cell distribution width RDW (%)**	13.23±0.85	13.3±0.83	13.53±1.13	13.88±1.10 *	13.76±1.42	**4.39, p=0.0357**
**Hemoglobin (g/L)**	138.00±18.28	149.38±21.26	145.63±23.49	138.95±16.53	147.63±25.81	1.37, p>0.2
**Mean corpuscular hemoglobin MCH (pg)**	28.90±1.50	28.88±1.56	29.06±1.48	28.69±1.14	28.85±1.48	1.17, p>0.3
**Mean corpuscular hemoglobin concentration MCHC (g/L)**	332.29±10.04	331.5±8.60	328.88±4.67	328.4±2.96	328.13±3.98	1.65, p>0.2
**Total platelet count (×10^9^/L)**	251.00±32.33	258.38±53.73	239.25±53.38	235.55±52.92	256.88±8.81	1.0, p>0.3
**Mean platelet volume (fL)**	8.4±0.76	9.13±1.16	9.6±1.26	8.82±0.73	8.6±0.73	**5.16, p=0.0162**

Comparing different stage of CDD treatments with Control 0D under Dunnett's multiple comparisons test

Significance * p<0.05, Sample size=8. Bold number indicated the detail significance of the value.

**Table 3:** Biochemistry in protein, lipids and glucose results under Flexible Abrosia facilitated Continual Dietary Deprivation for 14 days.

	Control 0D	Fasting 7D	Fasting 14D	Refeed 7-21D	Refeed 1-3M	One-way ANOVA F, *p* value
**Total protein(g/L)**	72.91±2.95	77.50±3.53	74.96±3.97	73.90±5.60	73.34±5.12	1.892, p>0.1
**Albumin (g/L)**	47.46±4.03	50.89±3.78 **	49.95±4.61	49.26±5.62	47.69±5.08	2..693, p=0.0968
**Globulin (g/L)**	25.46±2.058	26.65±4.31	25.01±3.09	24.64±2.52	25.66±2.44	1.285, p>0.3
**Alb vs Glb**	1.89±0.28	1.96±0.39	2.05±0.37 *	2.04±0.32 *	1.87±0.30	3.315, p=0.0739
**Urea nitrogen (mmol/L)**	4.83±1.53	3.34±1.89 *	3.08±1.76 **	4.35±1.97	4.42±1.58	**9.439, p=0.0005**
**Creatinine(µmol/L)**	64.82±16.95	73.00±19.41	73.25±18.58	63.60±15.76	64.43±13.17	**7.427, p=0.0066**
**Uric Acid(μmol/L)**	348.82±137.34	640.78±67.12 **	629.21±314.07 *	329.84±137.21	352.84±119.18	**13.42, p=0.0017**
**Myostatin (ng/L)**	12.73±5.67	6.92±3.98 *	6.63±2.28	8.31±3.58	9.05±4.00 *	**5.734, p=0.0145**
** **						
**Triglyceride(mmol/L)**	1.98±1.33	1.36±0.64	1.26±0.51	2.09±0.86	1.24±0.44	4.327, p=0.0519
**Lactic acid (mmol/L)**	2.24±0.47	2.28±0.84	2.25±0.47	1.85±0.44	2.22±0.42	1.114, p>0.3
**Lactate dehydrogenase(U/L)**	152.18±28.32	166.36±25.24	175.46±35.78 *	153.18±24.94	133.36±26.23	**6.866, p=0.0078**
**Total Cholesterol(mmol/L)**	5.71±0.97	6.59±1.34	5.91±1.59	5.80±1.08	5.71±1.23	2.793, p=0.0837
**High-Density Lipoprotein HDL(mmol/L)**	1.38±0.28	1.22±0.26	1.20±0.22	1.26±0.27	1.50±0.46	**3.976, p=0.0298**
**Low-Density Lipoprotein LDL(mmol/L)**	3.32±1.31	4.52±1.17	3.92±1.45	3.14±0.80	3.42±1.13	**6.374, p=0.0033**
**LDL/HDL**	2.46±0.99	3.98±1.71 *	3.48±1.74 *	2.55±0.72	2.58±1.39	**7.974, p=0.0025**
**Apolipoprotein A(g/L)**	2.08±0.29	1.70±0.46 *	1.66±0.40 ***	1.98±0.40	1.99±0.21	**6.879, p=0.0033**
**Apolipoprotein B(g/L)**	1.21±0.42	1.69±0.27 *	1.46±0.39	1.21±0.21	1.36±0.40	**5.818, p=0.0167**
**apoB/apoA**	0.59±0.22	1.12±0.56 *	0.96±0.47 *	0.62±0.08	0.71±0.31	**8.581, p=0.0132**
						** **
**Glucose (mmol/L)**	6.07±2.67	4.50±1.52 *	4.49±0.66	5.69±1.22	5.51±1.95	3.640, p=0.0745
**Insulin (µiu/ml)**	20.70±12.64	6.56±3.71 *	7.76±4.61	14.04±4.22	14.92±7.86	**7.520, p=0.016**
**HOMA-IR**	5.20±3.34	1.30±0.70 **	1.51±0.76 *	3.55±1.20	3.55±1.89	**12.42, p=0.0007**
**Glycosylated Hb (%)**	6.39±2.45	5.93±.244	5.72±1.94	5.92±1.70	5.80±1.56	2.33, p>0.1

Comparing different stage of CDD treatments with Control 0D under Dunnett's multiple comparisons test

Significance * p<0.05, ** p<0.01, *** p<0.001, Sample size n=8. Bold number indicated the detail significance of the value.

On the other hand, lipid related metabolites such as triglycerides showed a tendency to continuously decrease throughout the fasting period. Although there was no significant change in the concentrations of lactic acid, lactate dehydrogenase (LDH) increased significantly at 14^th^D-CDD. LDH is an enzyme that catalyzes the conversion of pyruvate into lactate (and vice versa) with concomitant interconversion of NADH and NAD+ which may indicate the constant fat depletion during fasting (**Table 3** and **Figure 3H**). The lipoprotein levels of HDL, LDL and Apolipoprotein (apo) A&B reached a plateau during the later stage of fasting and the results showed a similar trend of apoB & apoA which paralleled that of LDL & HDL. We observed an increased ratio of both LDL/HDL and apoB/apoA during the fasting period, but the ratios returned to normal after refeeding (**Table 3**). Meanwhile, β-hydroxybutyrate, a major ketone body metabolite, was significantly increased during the fasting period and gradually decreased after fasting was terminated (**Figure 3I**, F=20.83, p=0.0009, n=5).

### The change of metabolic or pathological factors during and after CDD

**Figure 4:**
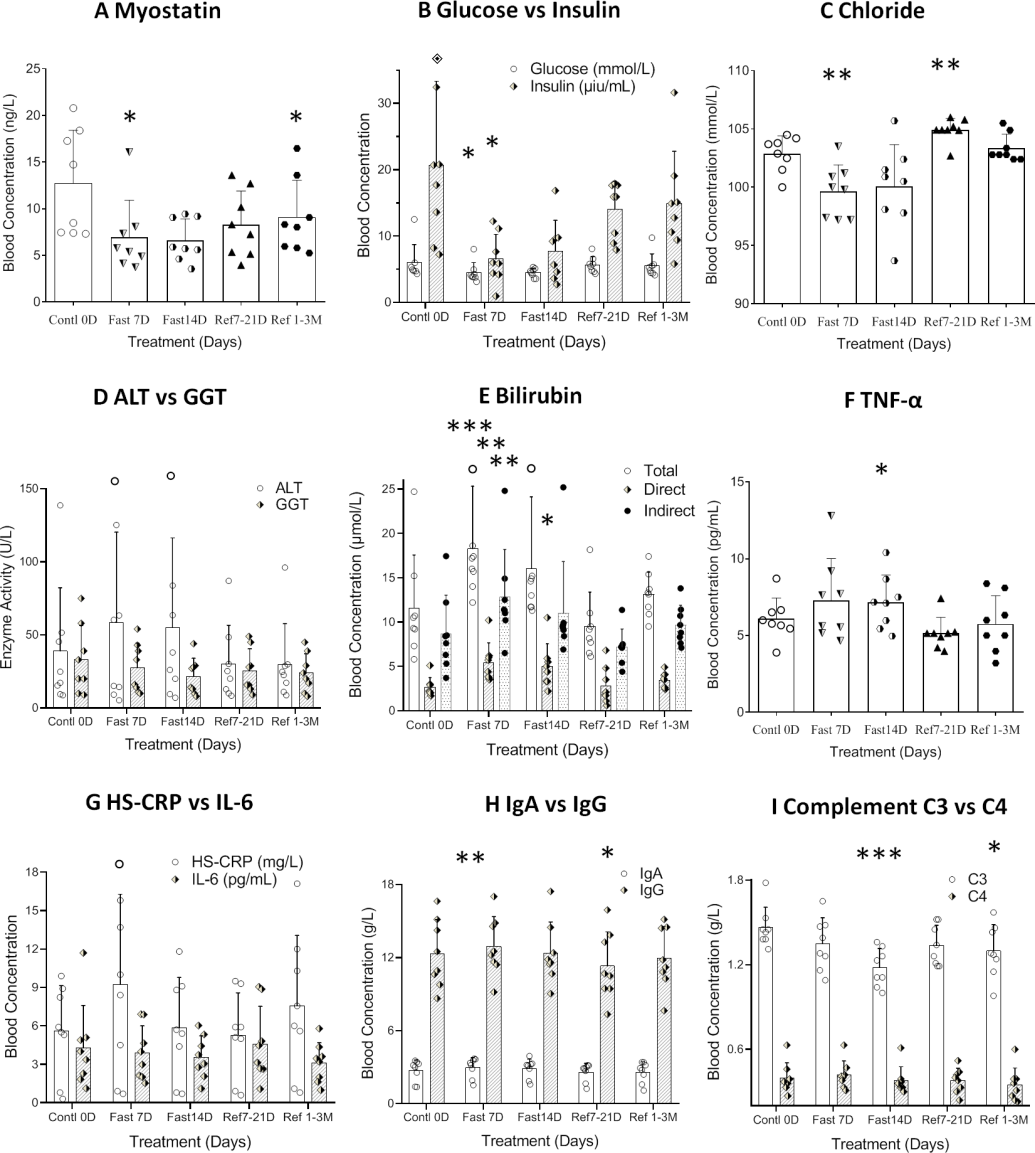
Differential analysis among 0D, 7D fasting, and refeeding results from either clinical laboratory tests or ELISA detection. Data were represented as MEAN ± SD in biochemistry and ELISA analysis with same range of treatment days in all experiment. The entire 14D’s fasting followed by 7D to 3mo’s refeeding were analyzed using repeated measure of one-way ANOVA on each column of variable. The data assignment is in the same was as described in **Figure 2**. Each treatment was analyzed with Tukey’s multiple comparisons test. Significance was assigned according to the description of GraphPad Prism: * p<0.05, ** p<0.01, *** p<0.001; the actual values are represented in the correspondent tables. **(A)**. Data were represented as results in MEAN ± SD of immunological analysis from ELISA analysis. **(B–I)**. Data were represented as results in MEAN ± SD of biochemistry analysis from hospital laboratory.

Carbohydrate metabolism throughout CDD also underwent significant changes. After 14 days of CDD, insulin levels were significantly down regulated (F=7.520, p=0.016, **Table 3**). Although glucose levels were not significantly reduced at 7^th^D among normal subjects, a significant reduction in insulin resistance (HOMA-IR) was observed (F=12.42, p=0.0007, **Table 3** and **Figures 4B, C**). Glycated hemoglobin (HbA1C), a form of hemoglobin which is commonly used in clinic to identify the three-month average plasma glucose concentration, also trended downwards, but failed to achieve statistical significance (p>0.1).

In order to explore the mechanism underlying the plateau of carbohydrate metabolism during longer term fasting, 8 trace cations and anions were investigated. Since the subjects took additional potassium and magnesium during fasting, the 2 cations failed to change significantly. Copper and iron showed various directional changes, but these failed to achieve statistical significance. The only ions that showed significant changes were chloride and zinc (**Table 4**, and **Figures 4D, E**). This data suggests that long-term fasting showed less impact on ion levels of the subjects and that metabolic regulation is able to compensate for changes in nutritional intake when undergoing long-term fasting.

**Table 4:** Biochemistry in mineral, age-related factors and enzymes results under Flexible Abrosia facilitated Continual Dietary Deprivation for 14 days.

	Control 0D	sFasting 7D	Fasting 14D	Refeed 7-21D	Refeed 1-3M	One-way ANOVA F, *p* value
**Copper (mmol/L)**	15.04±2.572	17.18±3.58	15.89±3.48	15.45±4.71	15.83±3.25	1.223, p>0.3
**Magnesium (mmol/L)**	0.90±0.10	0.93±0.13	0.96±0.096	1.01±0.064	0.94±0.085	1.482, p>0.2
**Iron (mmol/L)**	21.96±10.36	17.13±6.50	15.68±4.60	14.12±2.42	14.99±4.25	2.030, p>0.1
**Potassium (mmol/L)**	3.94±0.33	4.10±0.35	3.87±0.68	4.09±0.45	3.87±0.23	0.944, p>0.4
**Sodium (mmol/L)**	137.94±1.63	136.48±2.05	137.36±2.49	138.78±1.60	137.63±1.35	2.150, p>0.1
**Chloride (mmol/L)**	102.89±1.52	99.65±2.27 **	100.08±3.58	104.92±1.00 **	103.37±1.18	**10.56, p=0.0012**
**Calcium (mmol/L)**	2.33±0.078	2.41±0.087	2.42±0.10	2.63±0.77	2.84±0.78	0.855, p>0.4
**Zinc (µmol/L)**	13.47±3.71	14.54±4.451	17.30±5.86	11.35±5.74	11.56±4.45	**3.70, p=0.0336**
** **						
**Alanine aminotransferase (ALT) U/L**	38.92±43.37	58.38±62.07	55.00±61.44	30.27±26.39	29.87±27.85	3.493, p=0.0853
**Glutamic Oxalacetic Transaminase(U/L)**	29.60±14.32	40.73±26.12	40.60±24.74	23.52±8.18	24.60±8.33	3.667, p=0.0851
**Gamma-Glutamyl Transferase(U/L)**	33.00±23.51	27.50±16.89	21.63±12.41	25.55±14.96	24.00±14.19	3.611, p=0.0771
**Alkaline Phosphatase(U/L)**	66.88±19.90	75.00±25.70	73.38±28.47	63.18±22.29	67.14±22.46	1.505, p>0.2
**Total bilirubin(µmol/L)**	11.59±5.97	18.26±7.06 ***	16.01±8.11	9.54±3.83	13.13±2.51	**8.053, p=0.0073**
**Direct bilirubin(µmol/L)**	2.64±1.09	5.44±2.21 **	5.01±2.54 *	2.80±2.10	3.46±0.95	**10.44, p=0.0010**
**Indirect bilirubin(µmol/L)**	8.68±4.344	12.83±5.38 **	11.00±5.82	7.19±2.02	9.67±2.22	**4.929, p=0.0307**
** **						** **
**Tumor necrosis factor TNF-α (pg/mL)**	6.08±1.36	7.29±2.71	7.15±1.78 *	5.15±1.03	5.75±1.84	3.373, p=0.0710
**Insulin-like Growth Factors IGF-1 (ng/L)**	231.07±99.84	154.83±83.40	117.16±67.49	192.40±42.16	207.63±71.61	**4.688, p=0.0247**
**High-sensitivity C-reactive protein(mg/L)**	5.60±3.54	9.25±7.02	5.84±3.95	5.28±3.29	7.56±5.50	3.237, p=0.0804
**IL-6 (pg/mL)**	4.29±3.32	3.88±2.13	3.56±1.66	4.59±2.94	3.13±1.56	0.929, p>0.4
**Complement C3 (g/L)**	1.46±0.14	1.35±0.18	1.18±0.13 ***	1.34±0.14	1.30±0.19 *	**7.771, p=0.0021**
**Complement C4 (g/L)**	0.40±0.11	0.42±0.10	0.38±0.10	0.38±0.09	0.35±0.12	2.668, p>0.1
**IgA (g/L)**	2.73±0.87	2.98±0.82 **	2.89±0.78	2.59±0.73	2.60±0.86	**6.958, p=0.0039**
**IgG (g/L)**	12.32±2.80	12.93±2.44	12.38±2.56	11.32±2.81 *	11.98±2.56	**5.010, p=0.0168**
**IgM (g/L)**	1.10±0.65	1.22±0.88	1.09±0.69	1.06±0.63	1.11±0.50	0.9956, p>0.3

Comparing different stage of CDD treatments with Control 0D under Dunnett's multiple comparisons test

Significance * p<0.05, ** p<0.01, *** p<0.001, Sample size=8. Bold number indicated the detail significance of the value.

Alanine aminotransferase, glutamic oxalacetic transaminase, and alkaline phosphatase, which can stand in as readouts of liver function, showed a tendency towards increased activity during acute fasting, but prolonged fasting led to the opposite trend (**Table 4** and **Figure 4D**). Gamma-glutamyl transferase activity trended in the opposite direction (**Table 4**). Among the 8 subjects tested, only one subject showed abnormally high basal blood levels of bilirubin, and his total bilirubin level returned lower than normal range after 33 days’ recovery (CDD0D 24.7µmol vs Refed33D 15.40µmol). Bilirubin levels of other volunteers were all within the normal range. Based on the results of biochemistry analysis, CDD induced peak levels in most factors by the 7^th^ day, which might indicate some hydrolytic metabolism activated under autophagic stress during the start of fasting, which gradually tamps down at later stages of the fasting. After long-term recovery, most of factors mentioned above recovered toward their original levels (**Table 4** and **Figure 4E**).

Myostatin was originally identified as a new member of the transforming growth factor (TGF-ß) superfamily of signaling molecules, and its mRNA was detected almost exclusively in the skeletal muscle lineage. Studies revealed that loss of myostatin led to dramatic increases in skeletal muscle mass throughout the body, with individual muscles growing to about twice the normal size. Our results found that myostatin was not only significantly decreased during fasting, but also failed to recover during the entire refeeding recovery period. This result may indicate the importance of myostatin inhibition in modulating the balance between maintenance of skeletal muscle and fat during the prolonged fasting.

### The impact of anti-oxidative and inflammatory factors on physiological and biochemical effect of 14D-CDD

Antioxidant factors related with an anti-aging effect, such as Insulin-like growth factor type I (IGF-I), tumor necrosis factor (TNF-α), Interleukin 6 (IL-6), and C-reactive protein (HS-CRP) are all regarded as playing an important role in the onset of aging. IGF-I showed significant group reduction during fasting (F=4.688, p=0.0247), but IL-6 and TNF-α showed increase only at 7D-CDD and returned to normal levels after refeeding (**Table 4** and **Figures 4F, G**). There was a lack of statistical significance among the above 3 factors tested due to the limited sample size, and HS-CRP failed to show significance during and after CDD (**Table 4** and **Figure 4G**, p=0.0804).

One of the critical changes among inflammatory factors came from immunoglobulin IgA, IgG, IgM and Complement proteins C3 and C4. Levels of all of the 3 immunoglobulins tended towards a statistically significant increase, but only IgA showed significant increase at 7^th^D-CDD, and IgG showed a significant decrease only during the refeeding recovery stage from7-21D. The most interesting phenomenon was in Complement C3 levels, which showed a highly significant decrease at 14D-CDD, but also showed further decrease in longer-term refeeding recovery 1-3M (**Table 4** and **Figures 4H, I**). C3 is the most abundant complement protein in the body and circulating C3 is mostly made by liver hepatocytes. The lower Complement C3 levels after recovery from CDD might lead to reduce inflammatory reaction and confirm the healthy status of the hepatocytes and improve the other tissue’s function including skeletal muscle after experienced longer-term of fasting.

## Discussion

Our results demonstrated that a 14D-CDD fasting paradigm is a tolerable and efficient regimen, which might be very practical for long-term total fasting practice for general adoption. All subjects experienced reasonable body weight reduction (about 10% reduction during the full session of 14D-CDD) plus tolerable, moderate-intensity symptoms of starvation. Four of the subjects participated have accomplished detail self-estimation of experience in starvation, and the individuals and average score are summarized in **Figure 2I**. According to records from each subjects’ personal experience, although the administration of FA plus proper mineral supply at each mealtime enabled increased tolerance of hunger symptoms, the reports of some discomfort upon the onset of symptoms varied in larger range. Usually, the individual with over weighted body shape and potential metabolic syndrome such as abnormal of blood glucose level or higher blood pressure showed more tolerable to pangs of hunger.

In order to explore the metabolic abnormalities, bilirubin, a breakdown product of haemoglobin which is usually found during the breakdown of red blood cells and higher levels may indicate troubling in processing bilirubin into bile or increased rate of destruction of red blood cells. Usually, bilirubin serves as an endogenous antioxidant and marker of red blood cell turnover, as well gallbladder and liver function. We found significant increase in red blood cells distribution after 7D recovery from CDD (**Table 2** and **Figure 2H**). In fact, this result indicated beneficial effect after the CDD and was the only significant factor among all of the laboratory hematology results (**Table 2**). Total, direct and indirect bilirubin levels represent hepatobiliary function in clinic, and all showed significantly higher during CDD and completely returned to normal levels after recovery (**Table 4** and **Figure 4E**). Those biochemistry tests also indicated that certain enzymes or proteins related with bilirubin might be actively released during fasting to protect from autophagy induced catabolysis under stress (**Figure 4E**). We also investigated liver function analysis and found that alanine aminotransferase, glutamic oxalacetic transaminase, and alkaline phosphatase all showed a tendency towards increased activity during acute fasting, but prolonged fasting led to the opposite trend (**Table 4** and **Figure 4D**). Gamma-glutamyl transferase activity trended in the opposite direction (**Table 4**). Accordingly, levels of albumin increased at fasting 7D and gradually decreased to control level by the end of fasting and recovery. Albumin is a protein made by the liver which shows how well the liver is making certain proteins necessary for the body to fight infections and perform other functions. Therefore, bilirubin, liver function factors and protein levels reached higher but normal status during fasting and returned to normal by the end of CDD. We need to explore the subjects with original abnormal liver function to further analyze the detailed phenomenon which might relate with pang of hunger in the future.

In the initial fasting period (first 7D-CDD), subject’s BW dropped at the fastest rate (about 1~2lb/day). The speed of BW reduction turned to be moderate in later period of fasting (14D-CDD), which might indicate the system was safely buffered and protected from dramatic dehydration as long as subjects kept drinking plenty of liquid. In addition, the reduction in BW, BMI and the body fat still remained significant after the CDD has been terminated for more than a week or even up to 3 months (Refeed7-21D and Refeed1-3M, **Table 1** and **Figures 2A, B**). Comparing with the quickly returned to control level in both muscle and protein of BEIA after refeeding, it might indicate that continuously burned off fat which related with lighter gravity fat tissue than heavier protein. Eventually, it led to a plateau phenomenon of slowing down in BW reduction rate during later period of long-term fasting.

In order to clarify in more detail about the mechanism behind the observed phenomenon, we evaluated basal metabolic rate (BMR). Fasting and energy expenditure have been extensively studied, including the most recent prolonged fasting analysis which confirmed the consensus that BMR was well known of reduction during fasting and recover during refeeding (20). BEIA is an easy, non-invasive and fast medical device which works well in clinic among patients with stable water and electrolyte balance that is appropriate with regards to age, sex and race (21). Our study focuses on evaluating the practical value of FA-CDD, and therefore we adopted the simplest BEIA as a quick measurement on previous reported parameters (20). Instead of adopting sophisticated metabolic and biochemical equipment and procedures, we only focused on comparing with the relative differences in BMR values though BEIA to exclude systematic error rather than adopt time consuming and suffering analysis in the subjects through traditional BMR measurement during and after fasting.

We found that skeletal muscle mass reached the nadir of the response curve after fasting for 7 days(7D-CDD), and then showed no further down regulation by 14D-CDD measured by BEIA. However, body fat has shown to consistently decrease during the entire CDD procedure, even after long-term refeeding recovery from CDD. The changes in whole body total protein and body liquid content under BEIA show a similar pattern to that of skeletal muscle mass (**Table 3**, **Figures 2D–G**). When we further analyzed the blood protein vs lipid concentrations in biochemical assays, we realized that levels of protein showed a consistent expression pattern, but in opposite direction as shown in BEIA. This can be attributed to the fact that the total protein in BEIA represents whole body protein content, while total protein via biochemical measurement represents protein concentration in blood. Therefore, the two results can be seen to support each other (**Tables 2** vs **3**). Lipid and lipoprotein levels might be more complex. Although triglycerides tended to decrease in the blood level, it might indicate the increased tendency to utilize the lipids during fasting. Meanwhile lactic acid remained unchanged which might due to its importance as the nexus of a variety of metabolic pathways (**Table 3**). Cholesterol, which increased at Day 7 due to turnover observed during relative short-term fasting, returned to its original level by the end of 14D-CDD (**Table 3**). We also found that longer term recovery (1-3M) from CDD has returned most factors’ levels, which represented as stabilized metabolic status of both lipids and proteins in vessel after long-term recovery from CDD. However, the BEIA results indicated that the whole-body fat status has remained lower than control even after long-term recovery from CDD which indicated effective weight control benefit after prolonged fasting treatment (**Tables 1** vs **3**, and **Figures 2** vs **3**).

ApoA and apoB are regarded as correspondent cholesterol transporters of HDL and LDL metabolism, in which they act as transport vehicles to form lipoproteins and transport the lipids through lymphatic and circulatory systems. As such, the apoB/apoA ratio (correspondently as LDL/HDL) is a strong predictor of coronary heart disease risk (22). The results showed a paralleled trend of apoB vs apoA which was correspondent as that of LDL vs HDL. We observed an increased concentration of both LDL and apoB, which were correspondent as decrease in both HDL and apoA, during the fasting period, but both of the ratios returned toward normal after termination of CDD (**Table 3** and **Figure 3H**). The significant increase in both LDL/HDL and apoB/apoA during fasting indicated an increased risk of cardiovascular disease due to increase LDL and apoB by autophagy under fasting but reserve or even lowed HDL and apoA which might relate with systematic protection. In regarding with lipid factors, β-hydroxybutyrate, a major ketone body metabolite, was significantly increased during the fasting period, and gradually decreased after refeeding initiated (**Figure 3I**). These results supported the possibility that longer term fasting increased catabolism of lipid-related energy supplies (such as LDL and β-hydroxybutyrate) and reserve more health energy supplies such as HDL and triglyceride to further support ketone body and lipid metabolism, while enhancing clearance of damaged tissues through autophagy and proteolysis. Our result also indicated that apoB/apoA followed key roles in coordinating cholesterol transportation which led a further decreased ratio of LDL/HDL at later stage of fasting (**Table 3**, and **Figure 3I** at 14^th^D-CDD).

The U-shaped response curve might not always be a stereotypical of the factors under the effect of prolonged fasting. An extreme example is myostatin, which is a member of TGFβ family and acts as a negative regulator of muscle growth. Physiological aging is correlated with higher myostatin concentration (23), and myostatin inhibition has been applied in therapeutic treatment of sarcopenia (24). We found that myostatin baseline levels were significantly down regulated after fasting, below each subject’s own *ad libitum* level. Although refeeding led a slight rebound of myostatin, the level failed to return toward baseline even after refeeding recovery up to 3M (**Table 3**, **Figure 4A**). This evidence suggests a turnover of injured cells through autophagy during CDD, and this tissue renewal procedure is followed by the buildup of fresh muscle cells enabled by the low level of myostatin after refeeding. In addition, the corresponding changing patterns in muscle vs protein and fat vs lipid between BEIA and biochemistry readouts may explain the slower rate in BW changes, which might be due to the difference in the remaining muscle vs fat tissue proportion. In addition, the continued reduction of β-hydroxybutyrate after long-term recovery from CDD may indicate ongoing ketone body metabolism is maintained (**Figure 3I**). As a final result, the subjects showed a further reduction in both body fat and visceral fat area while the declining rate of muscle tissues was slowed down. Eventually, the total BW started to decline slower at CDD 14D than at CDD 7D. However, after 3 months’ recovery, as indicated in BEIA, the content of muscle mass might remain a higher than subject’s original fat level which indicated a more fitness body shape maintained during the refeeding period. Therefore, we recommended the subject maintain a ketogenic diet and restrict carbohydrate intake during initial two weeks of refeeding recovery period.

Most of the above results indicated a peak (or valley) around Day 7 of fasting, but also a relatively less or plateau shape of effect at 14^th^D during prolonged fasting (**Figures 1**–**4**). Of the factors tested, cholesterol, LDL, total protein, hemoglobin, lactate dehydrogenase, creatinine, uric acid, TNF-α, Cr, CK, ALT, and GOT concentrations all showed a tendency to peak in the blood. On the other hand, we found that triglyceride, HDL, glucose, insulin, BUN, and IGF-1 showed a tendency towards the lowest valley of concentration changes (**Figures 2**, **3**). Most of the factors that are up-regulated during CDD seem to relate with either adapting to decreases in energy or nutritional supply, or stress markers activated under nutrient depletion or clearance of the system during starvation. Those factors that were down-regulated during fasting were usually implicated in either alternative energy supply (such as proteins in whole body and hemoglobin) or critically reserved system consumption (such as BUN, triglycerides, and IGF-1). It seems that the system automatically chooses the beneficial and critical factors to preserve and selects the damaged elements to eliminate during total fasting, which might indicate that the well-controlled fasting-related autophagy is preferentially initiated and targeted upon damaged or unhealthy tissue.

Laurens have studied 10 days fasting among16 individuals under Buchinger Wilhelmi protocol with supplements of 200–250 kcal/day (19). Both reduction pattern of fat mass and lean soft tissues under fasting represented similar trend as our results. The increasing in 3-methyl-histidine at day 5 of fasting followed by decreasing confirmed the hypothesis of protein sparing follow early proteolysis under longer-term fasting. Our results applied a novel protocol with human unabsorbable calorie of 100 kcal/day and confirmed reservation of protein, especially muscle related tissues under 14D-CDD. We also established the possibility that this paradigm might apply with human unabsorbable prebiotics to prevent damage from intestinal flora and we found a phenomenon of turning point of CDD and the influence of myostatin, which made the protocol more practical in the human society.

Among the factors tested, only myostatin and C3 showed further decrease of activity at 14D-CDD, and the level of activity was not fully recovered even after 3M refeeding recovery (**Tables 3**, **4**, **Figures 4A, I**). Meanwhile, the subjective expression from each subject’s personal communications indicated obvious relief of hunger sensation after longer exposure of fasting (**Figure 2I**). Therefore, we found a turning point (TP) phenomenon, which might represent as the point in time where reciprocal transformation in both psychological, physical and biochemical parameters under CDD. Early studies from the last century indicated that glycine and valine doubled in the plasma during the first 7–10 days of starvation, and then slowly and progressively falls to a value below its overnight fasting value after 40 days of starvation (9). BUN rapidly decreases to 30% of its overnight concentration, then remained low during fasting which is a similar pattern to that of protein (**Figure 3B**). An early report also indicated that the concentration of creatinine in the blood slowly drifts downward, reflecting the decrease in muscle mass under starvation (9). However, our results did not support this phenomenon. Although there was a lack of significance of differences in creatinine levels through a column comparison due to insufficient sample size, the one-way ANOVA showed group significance, which indicated an efficiently preventing of muscle mass reduction (**Table 3** & **Figure 3C**).

Our results have confirmed the fact that the physiological system starts to convert to ketone body metabolism by increasingβ-hydroxybutyrate levels upon extended starvation. However, the levels of both triglycerides and β-hydroxybutyrate between 7^th^ and 14^th^ CDD showed almost no difference, and even tended to exhibit a plateau effect rather than further extension at the level throughout the duration of CDD (**Figures 3E–I**). The results in the changes of directions of both protein and lipid levels might indicate an establishment of alternative energy supply from ketone body metabolism, which eventually reduces proteolysis by the end of 14D-CDD. The TP concept seems to be of most physical and biochemical consequence during CDD, and it correlated with not only protein and lipid metabolism, but also the levels of other factors. In addition, according to the review by Owen and Hanson, the starvation studies from the last century usually lasted around 40 days (9). Our work restricted the CDD to 14 days, which ensured the safety of the protocol while maintaining efficacy. The key factor might be the TP, since once the fasting process passed the TP, the potential of self-healing ability was stimulated. However, ketone metabolism might NOT be only one of the benefits obtained by the subjects undergoing CDD, and therefore we will evaluate the paradigm in more detail in the future. Therefore, the TP concept would be further analyzed with more subjects and detailed time interval.

IGF-I, TNF-α and HS-CRP are all oxidative stress-induced protective factors, and the significance of TNF-α only at 14D-CDD might be due to variation of the data among subjects. Among the inflammatory factors tested, like most protein factors, IgA showed a highly significant increase at 7D-CDD, and then it remains stable during the recovery from fasting (**Table 4** and **Figure 4H**). IgA is the dominant immunoglobulin isotype produced in mammals, and is secreted across the intestinal mucosal surface. The function of IgA in host-microbial mutualism has depended mainly on indirect evidence of alterations in microbiota composition or penetration of microbes in the absence of somatic mutations (25). The deficiency of IgA is implicated in human primary immune deficiency, and it can exert metabolic control through the microbiota-gut-liver axis (26). IgG autoantibodies are directly pathogenic in myasthenia gravis and target neuromuscular junction proteins, causing neuromuscular transmission failure. Treatment approaches that reduce autoantibody levels, such as therapeutic plasma exchange and intravenous immunoglobulin, have been shown to be effective for IgM and IgG patients (27). Our result in showing a significant decrease in IgG only at early recovery of Ref7-21D indicated a beneficial effect on tissue building up after recovery from long fasting treatment (**Table 4** and **Figure 4H**). Both IgA and IgG are significantly influenced by prolonged fasting, but returned to normal levels just like most other factors measured. This might indicate that the effects are a consequence of immune response after nutritional treatment on starved intestinal flora. The nutrient deprivation during fasting might induce local inflammation, which resulted in increased IgA and possible changes in IgG as well due to class switching by B cells. However, neither IgA nor IgG has been specifically shown to influence tissue regeneration in healthy individuals, nor are these changes in serum Ig’s indicative of any physiological benefit/consequence.

IgG was only found significant reduction at Refeed 7-21D and Complement C3 was found significantly decreased at both 14D-CDD and after 1-3M refeeding recovery (**Table 4** and **Figure 4I**). From the degree of the response changes, it seems that C3 might be the most key indicators in regarding with the inflammatory changes under prolonged fasting. C3 is a crucial component of the innate immune system, and contributes to a major host mechanism for detection and clearance of potential pathogens. It is also important for pathogenesis of age-related macular degeneration (28). Compelling preclinical evidence established a role of adipose-tissue C3 and its cleavage products on acylation stimulating protein (ASP) in adipose tissue inflammation, insulin resistance and cardiometabolic diseases (29).

Our results suggest that the lowered levels of both C3 and myostatin after longer recovery from fasting which might imply some direct or indirect connection on the regulation of inflammatory response in regarding with skeletal muscle and hepatocyte activity during and after CDD. These results at least demonstrated that prolonged fasting might not be always detrimental in skeletal muscle atrophy. In fact, a well-controlled fasting paradigm might relate with a possible suppression in catabolism of liver during CDD and enhancement in tissue regeneration and strength after refeeding recovery. Under sufficient nutritional and exercise, the muscle is expected to recover at a faster rate through replacement with non-damaged, fresh tissues. Our other results also indicated long-term beneficial of liver function after repeated CDD practice. This hypothesis needs to be further analyzed by increasing the sample size and factories studied in order to precisely elucidate the mechanism behind the effects observed in this study.

## Conclusion

Regarding with the long-term fasting paradigms in human studies, there were academic reports that individuals who could survive without food from 3 to 63 days (9). These results have indicated that humans might have the ability to survive significantly longer without food than laboratory rodents. Basically, all of these studies were based on the extreme status of starvation in humans rather than the results from lab animals. The current study has applied an initial attempt to explore if such paradigms would be accepted in societal practice and be able to serve as a practical role in improving health of tissues and maintaining fitness.

Our novel dietary deprivation paradigm applied the administration of FA, a combination of herbal extracts, prebiotics and minerals supplements, and focused on a practical regimen to reduce the suffering from the practice of prolonged fasting. We also limited our maximum fasting time within 14 days. Our results suggested that the balance between protein and lipid corresponds to the proportion of skeletal muscle vs fat when fasting extended toward 7~14 days. As shown through the difference in proportions between muscle and fat during and after fasting, we have demonstrated the unique pattern of alterations in BW reduction after the TP and demonstrated the safety of this regimen under longer-term continuous dietary deprivation.

Our results also supplied the first evidence that, within the 14 days of fasting, the muscle-related protein catabolism starts to reach a plateau while lipocatabolism continued to increase. Our data supplied a possible clue that, after passing the TP, the system starts preventing further proteolysis and the protein levels are basically maintained at a set point. If a healthy individual follows this fasting regimen with extensive physical exercise and a high-protein diet, the existing muscle tissue will likely be frequently replaced with fresh muscle cells. Thus, our study outlines a more efficient and practical fitness-and-diet regimen that current routines implementing not only weight control, but also improves anti-aging effect and overall health through repeatedly application of carefully controlled prolonged fasting.

## Data availability statement

The original contributions presented in the study are included in the article/supplementary material. Further inquiries can be directed to the corresponding authors.

## Ethics statement

The studies involving human participants were reviewed and approved by the Ethics Committee of Henan University. The patients/participants provided their written informed consent to participate in this study.

## Author contributions

GDL, XXW and HXX designed the study and the trial. GDL, HXX, YQH and YF fully managed and performed the trials. YYL, XZ, XM, CLZ managed clinical monitor and care. XM collected and managed the data analysis. LLS, LW, YYW and YF managed the clinical laboratory analysis. YYY, SZ and SJH performed InBody 720 body composition analysis. QNW, YQH, and XM performed ELISA and other blood tests. GDL created and revised the manuscript, and is fully correspondent to the contents of the manuscript. All authors contributed to the article and approved the submitted version.
